# Supplementary material for: A new sensitive and fast assay for the detection of EGFR mutations in liquid biopsies
Source: PLoS One. 2021 Jun 24;16(6):e0253687. doi: 10.1371/journal.pone.0253687 (PMC8224962; doi:10.1371/journal.pone.0253687)
Supplement: S1 Table — S1A Table. Clinical-pathological characteristics of cohort I used for analyses by the TheraScreen® (QIAGEN), the Ion Torrent® (Thermo Fisher Scientific) and the SensiScreen® EGFR Liquid assay (PentaBase) platforms. Cohort I was collected at the Institute of Pathology in Locarno, Switzerland. Abbreviations: AC, adenocarcinoma; F, female; M, male. S1B Table. Clinical-pathological characteristics of cohort II used for analyses by the ctEGFR Mutation Detection Kit (EntroGen) and the SensiScreen® EGFR Liquid assay (PentaBase). Cohort II was collected at the Department of Pathology, Herlev-Gentofte University Hospital, Denmark. Abbreviations: AC, adenocarcinoma; F, female; M, male. (ZIP) [file pone.0253687.s001.zip › S1A_Table.docx]

| **Patient** | **Sex** | **Age** | **Tumor localization** | **Sample** |
| --- | --- | --- | --- | --- |
| 1 | F | 62 | Lung AC and bone/liver metastases | Plasma |
| 2 | M | 63 | Lung AC and bone metastases | Plasma |
| 3 | M | 71 | Lung AC and bone metastases | Plasma |
| 4 | M | 54 | Lung AC | Plasma |
| 5 | F | 77 | Lung AC and bone/brain metastases | Plasma |
| 6 | F | 54 | Lung AC and pleural metastases | Plasma |
| 7 | M | 49 | Lung AC and bone metastases | Plasma |
| 8 | M | 56 | Lung AC and bone/brain metastases | Plasma |
| 9 | M | 43 | Lung AC and bone/pleural metastases | Plasma |
| 10 | M | 53 | Lung AC and pleural metastases | Plasma |
| 11 | F | 37 | Lung AC and brain/pleural metastases | Plasma |
| 12 | F | 51 | Lung AC | Plasma |
| 13 | F | 71 | Lung AC and brain metastases | Plasma |
| 14 | F | 66 | Lung AC | Plasma |
| 15 | F | 84 | Lung AC and liver metastases | Plasma |
| 16 | M | 61 | Lung AC | Plasma |
| 17 | M | 74 | Lung AC and bone/brain metastases | Plasma |
| 18 | M | 66 | Lung AC and bone/pleural metastases | Plasma |
| 19 | M | 83 | Lung AC | Plasma |
| 20 | M | 71 | Lung AC | Plasma |
| 21 | M | 70 | Lung AC and pleural metastases | Plasma |
| 22 | M | 77 | Lung AC | Plasma |
| 23 | F | 51 | Lung AC and pleural metastases | Plasma |
| 24 | M | 63 | Lung AC | Plasma |
| 25 | M | 82 | Lung AC | Plasma |
| 26 | F | 64 | Lung AC | Plasma |
| 27 | F | 58 | Lung AC | Plasma |
| 28 | F | 73 | Lung AC | Plasma |
| 29 | M | 74 | Lung AC | Plasma |
| 30 | F | 65 | Lung AC | Plasma |
| 31 | F | 67 | Lung AC | Plasma |
| 32 | F | 58 | Lung AC and bone/liver metastases | Serum |
| 33 | F | 73 | Lung AC and bone metastases | Serum |
| 34 | M | 74 | Lung AC and bone metastases | Serum |
