# Supplementary material for: A new sensitive and fast assay for the detection of EGFR mutations in liquid biopsies
Source: PLoS One. 2021 Jun 24;16(6):e0253687. doi: 10.1371/journal.pone.0253687 (PMC8224962; doi:10.1371/journal.pone.0253687)
Supplement: S1 Table — S1A Table. Clinical-pathological characteristics of cohort I used for analyses by the TheraScreen® (QIAGEN), the Ion Torrent® (Thermo Fisher Scientific) and the SensiScreen® EGFR Liquid assay (PentaBase) platforms. Cohort I was collected at the Institute of Pathology in Locarno, Switzerland. Abbreviations: AC, adenocarcinoma; F, female; M, male. S1B Table. Clinical-pathological characteristics of cohort II used for analyses by the ctEGFR Mutation Detection Kit (EntroGen) and the SensiScreen® EGFR Liquid assay (PentaBase). Cohort II was collected at the Department of Pathology, Herlev-Gentofte University Hospital, Denmark. Abbreviations: AC, adenocarcinoma; F, female; M, male. (ZIP) [file pone.0253687.s001.zip › S1B_Table.docx]

| **Patient** | **Sex** | **Age** | **Tumor localization** | **Sample** |
| --- | --- | --- | --- | --- |
| 1 | M | 87 | Lung AC | Plasma |
| 2 | F | 74 | Lung AC | Plasma |
| 3 | M | 67 | Lung AC | Plasma |
| 4 | M | 69 | Lung AC | Plasma |
| 5 | M | 60 | Lung AC | Plasma |
| 6 | M | 57 | Lung AC | Plasma |
| 7 | F | 76 | Lung AC | Plasma |
| 8 | F | 44 | Lung AC | Plasma |
| 9 | F | 70 | Lung AC | Plasma |
| 10 | F | 75 | Lung AC | Plasma |
| 11 | F | 68 | Lung AC | Plasma |
| 12 | F | 78 | Lung AC | Plasma |
| 13 | M | 50 | Lung AC | Plasma |
| 14 | F | 67 | Lung AC | Plasma |
| 15 | F | 58 | Lung AC | Plasma |
| 16 | F | 73 | Lung AC | Plasma |
| 17 | M | 84 | Lung AC | Plasma |
| 18 | F | 75 | Lung AC | Plasma |
| 19 | F | 54 | Lung AC | Plasma |
| 20 | M | 89 | Lung AC | Plasma |
| 21 | F | 81 | Lung AC | Plasma |
| 22 | F | 71 | Lung AC | Plasma |
| 23 | F | 59 | Lung AC | Plasma |
| 24 | F | 67 | Lung AC | Plasma |
| 25 | F | 45 | Lung AC | Plasma |
| 26 | F | 65 | Lung AC | Plasma |
| 27 | M | 70 | Lung AC | Plasma |
| 28 | F | 74 | Lung AC | Plasma |
| 29 | F | 61 | Lung AC | Plasma |
| 30 | F | 66 | Lung AC | Plasma |
| 31 | M | 75 | Lung AC | Plasma |
| 32 | F | 80 | Lung AC | Plasma |
| 33 | F | 70 | Lung AC | Plasma |
| 34 | F | 45 | Lung AC | Plasma |
