# Supplementary material for: A new sensitive and fast assay for the detection of EGFR mutations in liquid biopsies
Source: PLoS One. 2021 Jun 24;16(6):e0253687. doi: 10.1371/journal.pone.0253687 (PMC8224962; doi:10.1371/journal.pone.0253687)
Supplement: S2 Table — Abbreviations: CDS, coding sequence; del, deletion; EGFR, Epidermal growth factor receptor; ins, insertion. (DOCX) [file pone.0253687.s002.docx]

| **Exon in EGFR** | **CDS mutation** | **Amino acid substitution** | **Cosmic ID** | **Well in E1 strip** |
| --- | --- | --- | --- | --- |
| **EGFR exon 18** | c.2156G>C | p.Gly719Ala | COSM6239 | B |
|  | c.2155G>A | p.Gly719Ser | COSM6252 | B |
|  | c.2155G>T | p.Gly719Cys | COSM6253 | B |
| **EGFR exon 19** | c.2240_2251del12 | p.L747_T751>S | COSM6210 | C |
|  | c.2239_2247del9 | p.L747_E749delLRE | COSM6218 | C |
|  | c.2238_2255del18 | p.E746_S752>D | COSM6220 | C |
|  | c.2235_2249del15 | p.E746_A750delELREA | COSM6223 | C |
|  | c.2236_2250del15 | p.E746_A750delELREA | COSM6225 | C |
|  | c.2235_2246del12 | p.E746_E749delELRE | COSM28517 | C |
|  | c.2239_2256del18 | p.L747_S752delLREATS | COSM6255 | C |
|  | c.2237_2254del18 | p.E746_S752>A | COSM12367 | C |
|  | c.2240_2254del15 | p.L747_T751delLREAT | COSM12369 | C |
|  | c.2240_2257del18 | p.L747_P753>S | COSM12370 | C |
|  | c.2239_2248>C (complex) | p.L747_A750>P | COSM12382 | C |
|  | c.2239_2251>C (complex) | p.L747_T751>P | COSM12383 | C |
|  | c.2237_2255>T (complex) | p.E746_S752>V | COSM12384 | C |
|  | c.2235_2255>AAT (complex) | p.E746_S752>I | COSM12385 | C |
|  | c.2237_2252>T (complex) | p.E746_T751>V | COSM12386 | C |
|  | c.2239_2258>CA (complex) | p.L747_P753>Q | COSM12387 | C |
|  | c.2239_2256>CAA (complex) | p.L747_S752>Q | COSM12403 | C |
|  | c.2237_2253>TTGCT (complex) | p.E746_T751>VA | COSM12416 | C |
|  | c.2238_2252>GCA (complex) | p.L747_T751>Q | COSM12419 | C |
|  | c.2238_2248>GC (complex) | p.L747_A750>P | COSM12422 | C |
|  | c.2237_2251del15 | p.E746_T751>A | COSM12678 | C |
|  | c.2236_2253del18 | p.E746_T751delELREAT | COSM12728 | C |
|  | c.2235_2248>AATTC (complex) | p.E746_A750>IP | COSM13550 | C |
|  | c.2235_2252>AAT (complex) | p.E746_T751>I | COSM13551 | C |
|  | c.2235_2251>AATTC (complex) | p.E746_T751>IP | COSM13552 | C |
|  | c.2237_2257>TCT (complex) | p.E746_P753>VS | COSM18427 | C |
|  | c.2237_2251del15 | p.L747_T751delLREAT | COSM23571 | C |
|  | c.2233_2247del15 | p.K745_E749delKELRE | COSM26038 | C |
|  | c.2234_2248del15 | p.K745_A750>T | COSM1190791 | C |
|  | c.2236_2248>CAAC (complex) | p.E746_A750>QP | COSM13557 | C |
|  | c.2232_2249del18 | p.K745_A750delKELREA | COSM221565 | C |
|  | c.2237_2253>TA (complex) | p.E746_T751>V | COSM133192 | C |
|  | c.2239_2257>T (complex) | p.L747_P753>S | COSM133197 | C |
|  | c.2239_2253>AAT (complex) | p.L747_T751>N | COSM51503 | C |
|  | c.2236_2259>ATCTCG (complex) | p.E746_P753>IS | COSM133191 | C |
| **EGFR exon 20** | c.2369C>T | p.Thr790Met (T790M) | COSM6240 | C |
|  | c.2303G>T | p.Ser768Ile | COSM6241 | E |
|  | c.2300_2301insCAGCGTGGA | p.D770_N771insSVD | COSM3728433 | D |
|  | c.2302_2303insCGCTGGCCA | p.A767_S768insTLA | COSM12425 | F |
|  | c.2307_2308ins15 | p.V769_D770insMASVD | COSM28638 | F |
|  | c.2307_2308insGCCAGCGTG | p.V769_D770insASV | COSM12376 | F |
|  | c.2308_2309insCCAGCGTGG | p.V769_D770insASV | COSM12426 | F |
|  | c.2308_2309insGGGTCGTGG | p.V769_D770insGVV | COSM18430 | F |
|  | c.2308_2309insGTT | p.D770>GY | COSM12427 | F |
|  | c.2309_2310AC>CCAGCGTGGAT | p.V769_D770insASV | COSM13558 | F |
|  | c.2310_2311insAGCGTGGAC | p.D770_N771insSVD | COSM85749 | F |
|  | c.2310_2311insGGCACA | p.D770_N771insGT | COSM1238029 | F |
|  | c.2310_2311insGGGTTT | p.D770_N771insGF | COSM655155 | F |
|  | c.2310_2311insGGT | p.D770_N771insG | COSM12378 | F |
|  | c.2310_2311insAACCCCCAC | p.H773_V774insNPH | COSM48920 | F+G |
|  | c.2310_2311ins9GCGTGGACA | p.D770_N771insSVD | COSM13428 | G |
|  | c.2316_2317insNNN | p.P772_H773insX | COSM21597 | G |
|  | c.2319_2320insAACCCCCAC | p.H773_V774insNPH | COSM12381 | F+G |
|  | c.2319_2320insCAC | p.H773_V774insH | COSM12377 | G |
|  | c.2319_2320insCCCCAC | p.H773_V774insPH | COSM12380 | G |
|  | c.2320_2321insCCCACG | p.H773_V774insAH | COSM1238028 | G |
|  | c.2321_2322insCCACGT | p.V774_C775insHV | COSM18432 | G |
|  | c.2322_2323insCACGTG | p.V774_C775insHV | COSM22948 | G |
| **EGFR exon 21** | c.2573T>G | p.Leu858Arg | COSM6224 | H |
|  | c.2573_2574TG>GT | p.Leu858Arg | COSM12429 | H |
|  | c.2582T>A | p.Leu861Gln | COSM6213 | D |
