# Supplementary material for: A new sensitive and fast assay for the detection of EGFR mutations in liquid biopsies
Source: PLoS One. 2021 Jun 24;16(6):e0253687. doi: 10.1371/journal.pone.0253687 (PMC8224962; doi:10.1371/journal.pone.0253687)
Supplement: S3 Table — Abbreviations: Ct, Cycle threshold; EGFR, Epidermal growth factor receptor. (DOCX) [file pone.0253687.s003.docx]

|  | **EGFR exon 19 deletions** | | **EGFR L858R** | | **EGFR T790M** | |
| --- | --- | --- | --- | --- | --- | --- |
| **Range of mutated template added** | 0.1-50 % | 1-50 % | 0.1-50 % | 1-50 % | 0.1-50 % | 1-50 % |
| **Equation** | y = -3.60x + 39.89 | y = -3.67x + 39.95 | y = -2.58x + 38.42 | y = -3.03x + 39.38 | y = -3.14x + 39.21 | y = -3.51x + 39.98 |
| **R^2^** | 0.9669 | 0.9902 | 0.9812 | 0.9986 | 0.9866 | 0.9978 |
