# Supplementary material for: A new sensitive and fast assay for the detection of EGFR mutations in liquid biopsies
Source: PLoS One. 2021 Jun 24;16(6):e0253687. doi: 10.1371/journal.pone.0253687 (PMC8224962; doi:10.1371/journal.pone.0253687)
Supplement: S4 Table — Abbreviations: Ct, Cycle threshold; EGFR, Epidermal growth factor receptor, SD, Standard deviation. (DOCX) [file pone.0253687.s004.docx]

| **Template Concentration (%)** | **EGFR T790M** | | **EGFR L858R** | | **EGFR Exon 19 deletion** | |
| --- | --- | --- | --- | --- | --- | --- |
|  | Ct Average | Ct SD | Ct Average | Ct SD | Ct Average | Ct SD |
| 50 | 29.86 | 0.08 | 32.20 | 0.22 | 29.86 | 0.09 |
| 10 | 32.25 | 0.04 | 34.09 | 0.39 | 32.16 | 0.14 |
| 5 | 33.12 | 0.05 | 34.93 | 0.33 | 33.41 | 0.19 |
| 2 | 34.78 | 0.46 | 36.28 | 0.67 | 34.70 | 0.56 |
| 1 | 35.80 | 0.52 | 36.60 | 1.19 | 36.55 | 0.37 |
| 0.5 | 36.63 | 0.19 | 37.09 | 0.62 | 37.68 | 0.49 |
| 0.1 | 38.05 | 0.51 | 37.11 | 0.56 | 38.55 | 0.64 |
| 0 | -- | - | - | - | - | - |
