# Supplementary material for: A new sensitive and fast assay for the detection of EGFR mutations in liquid biopsies
Source: PLoS One. 2021 Jun 24;16(6):e0253687. doi: 10.1371/journal.pone.0253687 (PMC8224962; doi:10.1371/journal.pone.0253687)
Supplement: S5 Table — Ct values obtained when challenging the assays with either cell lines harbouring EGFR mutations or plasmid containing various mutated EGFR sequences diluted in WT cfDNA. Templates are analyzed in duplicates on the reference assay and in 4-double determination on the mutation specific assays. Abbreviations: Ct, Cycle threshold; EGFR, Epidermal growth factor receptor; NA, not available. (DOCX) [file pone.0253687.s005.docx]

| **Template** | **EGFR T790M** | | **EGFR L858R** | | **EGFR Exon 19 deletion** | |
| --- | --- | --- | --- | --- | --- | --- |
|  | Reference/Ct | Assay/Ct | Reference/Ct | Assay/Ct | Reference/Ct | Assay/Ct |
| H1975 (5 ng) | 31.05 | 31.80 | 31.82 | 32.36 | 31.53 | - |
| H1975 (5 ng) | 30.99 | 31.69 | 31.77 | 32.78 | 31.19 | - |
| H1975 (5 ng) | NA | 31.90 | NA | 32.62 | NA | - |
| H1975 (5 ng) | NA | 31.73 | NA | 32.60 | NA | - |
| H1650 (5 ng) | 28.77 | - | 29.71 | - | 29.69 | 29.47 |
| H1650 (5 ng) | 29.12 | - | 29.97 | - | 29.70 | 29.40 |
| H1650 (5 ng) | NA | - | NA | - | NA | 29.20 |
| H1650 (5 ng) | NA | - | NA | 43.61 | NA | 29.63 |
| EGFR G719D + human gDNA (5 ng) | 29.87 | 41.76 | 30.75 | - | 30.63 | - |
| EGFR G719D + human gDNA (5 ng) | 29.47 | - | 30.85 | - | 31.01 | - |
| EGFR G719D + human gDNA (5 ng) | NA | - | NA | - | NA | - |
| EGFR G719D + human gDNA (5 ng) | NA | - | NA | - | NA | - |
| EGFR L861Q + human gDNA (5 ng) | 30.41 | - | 30.21 | 43.79 | 30.18 | - |
| EGFR L861Q + human gDNA (5 ng) | 29.52 | - | 29.96 | 41.00 | 29.73 | - |
| EGFR L861Q + human gDNA (5 ng) | NA | - | NA | 42.54 | NA | - |
| EGFR L861Q + human gDNA (5 ng) | NA | - | NA | 41.21 | NA | - |
| EGFR L747S + human gDNA (5 ng) | 29.90 | - | 30.71 | - | 28.54 | 43.54 |
| EGFR L747S + human gDNA (5 ng) | 30.04 | - | 30.87 | - | 28.28 | - |
| EGFR L747S + human gDNA (5 ng) | NA | - | NA | - | NA | - |
| EGFR L747S + human gDNA (5 ng) | NA | - | NA | - | NA | - |
