# Supplementary material for: A new sensitive and fast assay for the detection of EGFR mutations in liquid biopsies
Source: PLoS One. 2021 Jun 24;16(6):e0253687. doi: 10.1371/journal.pone.0253687 (PMC8224962; doi:10.1371/journal.pone.0253687)
Supplement: S6 Table — Abbreviations: Ct, Cycle threshold; EGFR, Epidermal growth factor receptor. (DOCX) [file pone.0253687.s006.docx]

|  | **Template** | **Ct Statistics** | | |
| --- | --- | --- | --- | --- |
|  |  | **Average±SD** | **Lower 26.5% fractile** | **Upper 36.8% fractile** |
| **Exon 19 deletion** | c.2235-2249del (Glu746-Ala750del) | 38.63±0.76 | 38.38 | 39.56 |
| **T790M** | c.2369C>T (p.T790M) | 38.18±0.86 | 37.61 | 38.40 |
| **L858R** | c.2573T>G (p.L858R) | 37.43±0.95 | 37.04 | 38.30 |
