# Supplementary material for: A new sensitive and fast assay for the detection of EGFR mutations in liquid biopsies
Source: PLoS One. 2021 Jun 24;16(6):e0253687. doi: 10.1371/journal.pone.0253687 (PMC8224962; doi:10.1371/journal.pone.0253687)
Supplement: S7 Table — S7A Table. EGFR phenotypes obtained for cohort I as detected by the TheraScreen® (QIAGEN), the SensiScreen® EGFR Liquid assay (PentaBase) and the Ion Torrent® (Thermo Fisher Scientific) platforms. Abbreviations: del, deletion; EGFR, Epidermal growth factor receptor; IOT, Ion Torrent methodology; WT, wild type. aFor one of the three samples (Sample 11) with this combinatorial phenotype, using 9 ng of cfDNA as input for the ctEGFR Mutation Detection Kit (EntroGen) did only detect the EGFR exon 19 deletion variant, not the p.T790M variant. Triplicate measurements using 18 ng of cfDNA as input per PCR reaction and absolute quantification resulted in detection of the p.T790M variant in addition to the exon 19 deletion variant. From the standard curve, it was estimated that the copy number of the p.T790M variant was at the level 1–2 copies in 18 ng of cfDNA input. The SensiScreen® EGFR Liquid assay (PentaBase ApS) simplex did detect the exon 19 deletion using 9 ng of cfDNA per PCR reaction. bFor two of the three samples (Sample 21 & 22) with this combinatorial phenotype, the DNA input amount available was too low for detection of the EGFR p.T790M variant by the SensiScreen® EGFR Liquid assay (PentaBase ApS) as previously evaluated from the low copy number of p.T790M detected at higher DNA input amounts by the ctEGFR Mutation Detection Kit (EntroGen). Hence, these two samples should be considered as agreement samples, both detecting the exon 19 deletion. cWhile the ctEGFR Mutation Detection Kit (EntroGen) detected an EGFR exon 19 deletion, the p.T790M variant but not the exon 19 deletion was detected by the SensiScreen® EGFR Liquid assay (PentaBase) (Sample 31). Duplicate measurements using the ctEGFR Mutation Detection Kit (EntroGen) showed two amplification curves for the p.T790M variant however below the threshold. S7B Table. Overview of the EGFR gene status for each plasma sample in cohort II as detected by the Entrogen ctEGFR Mutation Detection Kit (EntroGen) and the [file pone.0253687.s007.zip › S7A_Table.docx]

| **Patient** | **TheraScreen^®^**  **(QIAGEN)** | **SensiScreen^®^ EGFR Liquid assay (PentaBase ApS)** | **IOT^®^ Oncomine cell-free nucleic acids assay (Thermo Fisher Scientific)** |
| --- | --- | --- | --- |
| 1 | T790M + Exon 19 del. | T790M + Exon 19 del. | T790M + Exon 19 del. |
| 2 | T790M + L858R | T790M + L858R | T790M + L858R |
| 3 | T790M + Exon 19 del. | T790M + Exon 19 del. | T790M + Exon 19 del. |
| 4 | T790M + Exon 19 del. | T790M + Exon 19 del. | T790M + Exon 19 del. |
| 5 | T790M + Exon 19 del. | T790M + Exon 19 del. | T790M + Exon 19 del. |
| 6 | T790M + Exon 19 del. | T790M + Exon 19 del. | T790M + Exon 19 del. |
| 7 | T790M + Exon 19 del. | T790M + Exon 19 del. | T790M + Exon 19 del. |
| 8 | T790M + Exon 19 del. | T790M + Exon 19 del. | T790M + Exon 19 del. |
| 9 | Exon 19 del. | Exon 19 del. | Exon 19 del. |
| 10 | Exon 19 del. | Exon 19 del. | Exon 19 del. |
| 11 | Exon 19 del. | Exon 19 del. | Exon 19 del. |
| 12 | WT | WT | WT |
| 13 | WT | **L858R** | **L858R** |
| 14 | WT | **L858R** | **L858R** |
| 15 | WT | WT | WT |
| 16 | WT | WT | WT |
| 17 | WT | WT | WT |
| 18 | WT | WT | WT |
| 19 | WT | WT | WT |
| 20 | WT | WT | WT |
| 21 | WT | WT | WT |
| 22 | WT | WT | WT |
| 23 | WT | WT | WT |
| 24 | WT | WT | WT |
| 25 | WT | WT | WT |
| 26 | WT | WT | WT |
| 27 | L858R | L858R | L858R |
| 28 | T790M + L858R | T790M + L858R | T790M + L858R |
| 29 | Exon 19 del. | Exon 19 del. | Exon 19 del. |
| 30 | L861Q | L861Q | L861Q |
| 31 | T790M | T790M | T790M |
| 32 | T790M + Exon 19 del. | T790M + Exon 19 del. | T790M + Exon 19 del. |
| 33 | T790M + L858R | T790M + L858R | T790M + L858R |
| 34 | T790M + Exon 19 del. | T790M + Exon 19 del. | T790M + Exon 19 del. |
