# Supplementary figures and images for: A new sensitive and fast assay for the detection of EGFR mutations in liquid biopsies
Source: PLoS One. 2021 Jun 24;16(6):e0253687. doi: 10.1371/journal.pone.0253687 (PMC8224962; doi:10.1371/journal.pone.0253687)

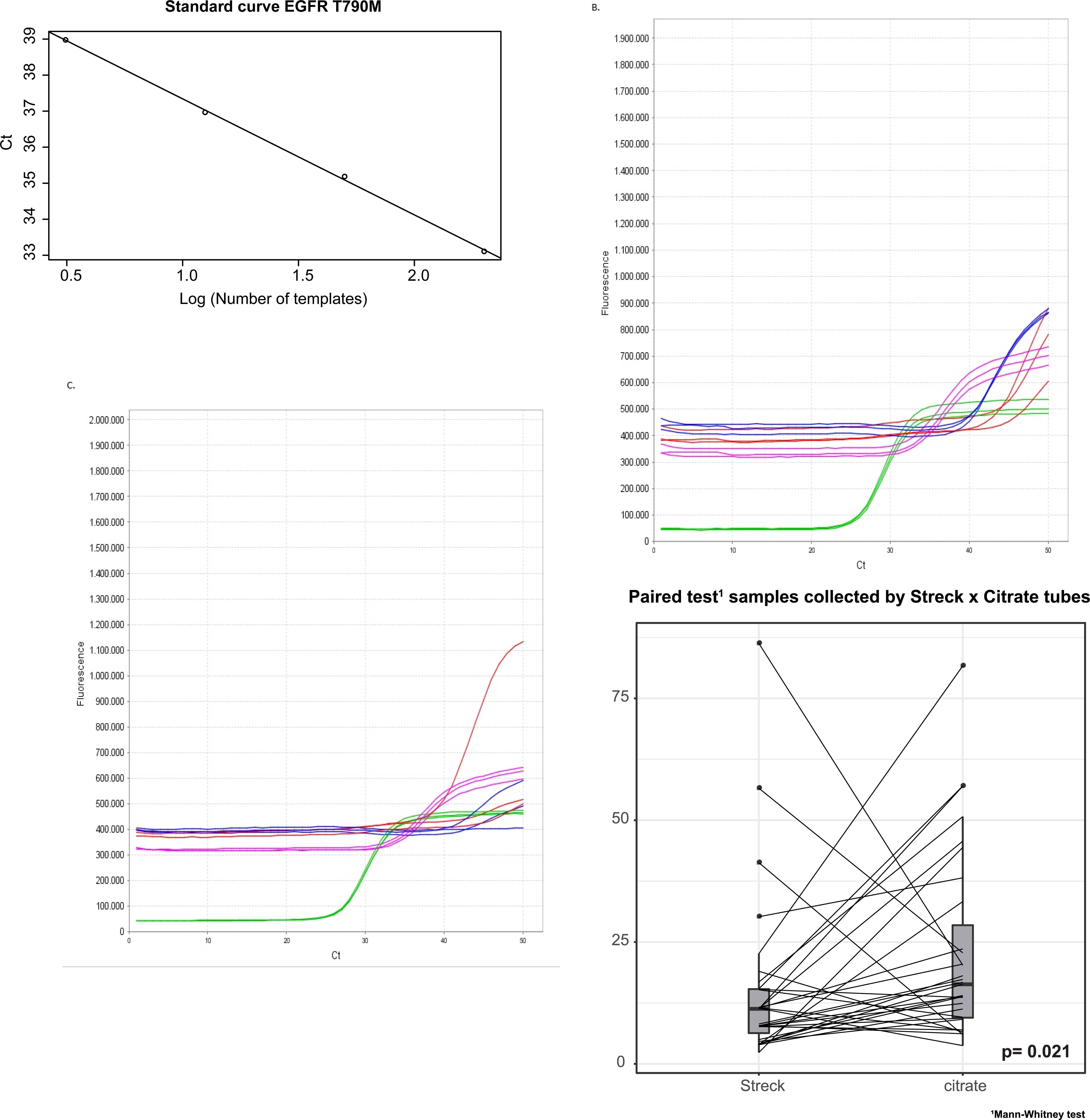

Supplement: S1 Fig — A. Standard curve for the EGFR p.T790M target (EntroGen) constructed using the positive control provided with the ctEGFR Mutation Detection Kit (EntroGen). Four-fold template copy number differences was assessed using a six-log-scale ranging from theoretical 0,195 copy to 200 copies. B. Multicomponent plot of triplicate measurements for sample 11 (Cohort II) using 18 ng of cfDNA per PCR. As evaluated from time of detection (B) and compared to the standard curve (A), the absolute DNA copy numbers of the EGFR c.2369C>T (p.T790M) variant (Blue amplification curves) were estimated to be in the range 1–2 copies in 18 ng of cfDNA. C. Multicomponent plot of triplicate measurements for sample 11 (Cohort II) using 9 ng of cfDNA per PCR. Decreasing the amount of cfDNA to 9 ng confirmed that the copy numbers of the EGFR c.2369C>T (p.T790M) variant (Blue) was low for sample 11 (Cohort II) using 18 ng of cfDNA (B). B. and C.: Internal control primers amplify the beta-2 microglobulin (B2M) DNA in samples and are used both to determine the condition of reagents and whether the reaction contains sufficient amount of amplifiable DNA (Green amplification curves). Detection of EGFR exon 19 deletion variant (Purple amplification curves). Non-specific detection by the EGFR c.2573T>G p.L858R target (Red curves). Abbreviations: Ct, Cycle threshold; EGFR, Epidermal growth factor receptor. D: Results of the comparison between cfDNA extracted from blood collected from two different tubes (streck vs citrate). A difference was observed between those two extraction methods (p = 0.021), with plasma citrate yielding higher amounts of DNA. (TIF) [file pone.0253687.s008.tif]
