# Supplementary material for: A new sensitive and fast assay for the detection of EGFR mutations in liquid biopsies
Source: PLoS One. 2021 Jun 24;16(6):e0253687. doi: 10.1371/journal.pone.0253687 (PMC8224962; doi:10.1371/journal.pone.0253687)
Supplement: Attachment — Submitted filename: Review Jensen et al, A new sensitive and fast assay for the detection of EGFR mutations in liquid biopsies.docx [file pone.0253687.s009.docx]

Comments in the review of Jensen et al, A new sensitive and fast assay for the detection of EGFR mutations in liquid biopsies

The authors have presented a paper which is important in further defining performance of the tools available for aiding physicians in applying targeted therapy to their patients. This paper explores analytical measures allowing the end user a way to understand how the test performs and to be able to assess how the test compares to other options an end user has available. In defining the performance of the test, the authors explore analytical measures such as limit of detection, linearity, repeatability and accuracy as approached through orthogonal comparison to two other ctDNA platforms. This paper follows the content of Riva et al, SensiScreen® KRAS exon 2-sensitive simplex and multiplex real-time PCR-based assays for detection of KRAS exon 2 mutations, PLoS One 2017 and the reviewer recognizes that the Riva paper’s approach and presentation has been accepted prior. However, there are some items which this reviewer feels need further consideration by the authors. The comments and suggestions are offered in order to harmonize the terminology in describing the analytical performance of diagnostic tests thereby allowing physicians/readers who are assessing testing methods as published in peer reviewed journals to see measurements based on clear and consistently applied definitions of analytical performance.

1) General comment: With your data, please include appropriate errors or ranges within the data you present. For instance, with your first plot showing the age of the patients in cohorts, what is the mean, range and did you apply a statistical measure to show they are the same? For the dilution series, what is the CV or SD experienced at each dilution point. Etc.
2) In the use of contrived material to define the analytical performances of the test the authors did not indicate that fragmented DNA was utilized in these experiments. If fragmentation was used, please indicate the process (sonication, restriction enzymes etc) and mention or perhaps show in the appendix that your fragmentation procedure produced a range of fragments somewhat similar to what is found from cfDNA. This relates to both the cell line material and the Promega mixed human genomic DNA which is sold as having >90% of the DNA as longer than 50kb in size.

If the authors did not utilize fragmented DNA then they should defend why this diagnostic approach (SensiScreen) would not be impacted by random fragmentation which could break DNA between the For and Rev primers thereby allowing the proper non-binding of the BaseBlocker in a fragment with the mutation and allowing binding of the For primer but extension stopping prior to where proper binding of the Rev primer would occur. This is important in the claims being made in the paper regarding the analytical sensitivity of the diagnostic system. Assuming no fragmentation of your cell line DNA/human DNA, are there other impacts on the PCR reaction which may be influenced by larger vs fragmented DNA besides the random fragmentation within the amplicon region? Ideally a contrived analyte should mimic as best as possible the biological analyte and if this is not important please defend why the authors chose to this route.

3) The Authors utilize the term LoD (limit of detection) in a manner which is not the acceptable application of this term in the description of the analytical limits for a clinical assay (for example CLSI-EP17A or IUPAC Recommendations (L Currie, Nomenclature in evaluation of analytical methods including detection and quantification capabilities (IUPAC Recommendations 1995) ACA 391, 105-126).
   a. For assessing the assay’s background/noise, the authors used only one sample (WT genomic DNA). With the lack of using age matched normal donors (samples assumed to not have the mutation present) with DNA isolated from plasma in describing the background (limit of blank) for each variant in the assay, the readers to cannot be assured that the claim of being able to detect 1 copy is in actuality the clear detection of the signal arising from one copy of the target DNA above what would be experienced with samples without the mutation tested under the same conditions (input level etc).

Authors should supply evidence supporting the limit of blank and also provide the limit of detection as defined by guidelines. Often this is defined in relationship to LoB and where 95% of the measurements is a repeated sample fall above the LoB. It can be acceptable to defend the success rate at a lower value, ie LoD90, but it should be clear to the reader what definition is used for LoD.

Should the authors move away from the use of the term LoD and describe this as something like the “lowest value detectable” by the assay system, the lack of evidence from the testing of samples/donors without the given mutation raises questions as to whether or not the detection of 1 copy is limited to only samples/patients who carry the given variant and should be addressed.

4) The above discussion on LoB leads to another deficiency in this paper and that is in establishment of the specificity of the test. It was shown in the post-hoc analysis of the data from the gefitinib pivotal trial by Mok et al (NEJM 2009) that the very worst outcome for a patient being considered for EGFR TKI usage or for SOC-doublet chemo, that a false positive EGFR mutation call would lead the patient to not receive doublet therapy and be put on TKI therapy thereby receiving a therapy with a detrimental survival outcome. The authors should consider running a set of real plasma samples (age matched, if possible at the same DNA input levels as your contrived cell line dilutions) to both get a sense of test specificity and to establish the LoB, thereby defending that your ability to detect down to 1 copy is well above any noise in the system. Running a single wt Promega DNA mixture is not sufficient for this.
5) In cohort I which describes the use of Streck BCT tubes, the authors refer to serum in addition to plasma. It is unacceptable for blood being processed to serum to be held for 48 hours prior to separation as leukocyte degradation and subsequent release of leukocyte genomic DNA has been shown to occur. Please clarify if serum is the correct term here and if it was, further description of the use of serum tubes and a discussion on the impact of contaminating genomic DNA should be offered by the authors.
6) Results section
   a) Sensitivity – please provide the CV or SD at each dilution point.
   b) Linearity – R2 describes the relationship between two measured variable and the strength of the relationship. 0.97 means that you show a very good linear relationship between ct and the estimated copy number but you do not show linearity as have referred to this as. It would be more appropriate to apply other tests of linearity to your dilution experiment if you wish to claim or show that your test has linearity. I would recommend that you read CLSI guidance EP-06. Applying an assessment of a 1^st^ order polynomial fit to this data to a second or third order polynomial fit will show where the data is linear and where the linearity drifts.
   c) Repeatability – Section well written and described. Please describe the data further as you see fit. Example mean, SD, 10^th^ and 90^th^ percentile etc. .
7) SensiScreen in clinical samples
   a) You give median DNA/ml extracted, please include 10^th^ and 90^th^ percentile or range in the text.
   b) To follow on from a comment above regarding serum in cohort 1, I see you have 3 serum samples. Here or later in the discussion if appropriate, you may wish to comment that the DNA/ml isolated from the serum was within the range of the Streck plasma suggesting that a gross contamination from DNA released from leukocytes was not observed.
   c) For data in figure 4 please perform a statistical test to compare the ng cfDNA from Streck vs citrate. It appears that citrate may give a statistically higher amount of DNA. Please test if the two sets of isolated DNA are different from one another. One might assume that there is a release of HMW DNA in the citrate cohort and you should discuss if this HMW DNA could have an impact on the assessment of the mutations by any methodology. Worth considering this in your discussion should they show a statistical difference..
   d) For table 1A and 1B it is rather unusual to see T790M without the 19del or other driver such as L858R. This indicates that the patient has an inherited T790M (unusual but does occur) or that the driver is not seen due to the tests chosen not having the designs for the driver mutation. There are 5 total examples of this. I see that you discuss this example “c”. You are saying that EntroGen detected this sample as ex19del positive but T790M negative and SensiScreen detected T790M but not the ex19Del. This is biologically very unusually as T790M is a branch variant from the ex19del truncal variant. Ex19del is observed in quantitative tests as having a higher VAF than the acquired mutation. Using table S3b, for the other examples where T790M is detected without the driver detected, can you describe why this might have occurred? Is or could the ex19del be an unusual deletion which SensiScreen did not have a design for? How do you defend not seeing the drivers from patient 25,26 and 27? What is the ct of the T790M for these samples? Are they just at the 1 copy area? Including the ct values in the supplemental tables would help a reader to further understand your observations.
   e) In table S3A and S3B it would be ideal if you also included the measured values (ct, VAF, copy number; whatever is the usual output) for all liquid biopsy tests.
   f) In Table S3A, you should identify the construct of the 19del detected in Oncomine as 19del comes in many presentations. In table S3B, it is unclear what tissue test was used to confirm the variants, however if it is available, please include the construct of the 19del. It is important to know if there is a design available for the specific ex19del in the liquid biopsy tests.
   g) Patient 13 cohort II was WT in tissue and now has only T790M in both liquid assays. Was this patient treated with 1^st^ or 2^nd^ gen TKI? Why were they treated with TKI if no evidence of EGFR driver mutation? How did a T790M arise?
   h) For table 1A and 1B, in accurate NGS testing, the EGFR driver is nearly always found at greater VAF than the acquired T90M mutation. It is important that when you compare the two tests that you do discuss the impact of different DNA amounts if the DNA amounts for the testing was limited due to the need to split the samples up for this study. Your discussion regarding the ex19 variant detected only at higher levels of DNA input for EntroGen vs SensiScreen is a good example of detection sensitivity vs sensitivity with increasing input.
   i) Table IB, footnote (b), and paragraph starting on line 394 “For cohort II, as expected from the results….” In order to justify your conclusion more clarification is necessary. You say that SensiScreen only had limited material hence the T790M dropped out but you wish to consider these as “in agreement” I need a bit more information before I would be in agreement. It appears to be a discordance which results in dire clinical guidance. For prescribing osimertinib after 2^nd^ gen EGFR TKI, one must have a T790M mutation and these results with SensiScreen deny the patient the chance to start on osimertinib. My question is regarding the limited material: did the amount used in this sample pass the reference gene QC requirement for the test? I believe this is a ct of the reference to be between 29 and 40 as I read in the “SensiScreen, instructions for use”. Or did it show lower than a 40 ct? If yes, it was lower than the low limit in the package insert I can see your argument and I would describe it this way. However, if the reference passed QC / deemed adequate, and thus the sample was acceptable, there is a need to discuss this beyond “we consider them in agreement”. Should the manufacturer allow for a very low level of amplifiable DNA in the test or DNA input for the test, this should be a critique in your discussion part of your paper that a test can be valid but allowable levels my limit the detection to up to 10% VAF or such. By not having a critical lower limit of input, a doctor relying on the results of this test for aiding patient care would provide inconsistent patient care.
   j) To expand further on the reference measurement above, another important consideration in your data is following the limits set by the manufacturer for the test. Part of the sample QC is in the use of a reference target which “is used to assess the amount of amplifiable DNA in the sample”. A sample is considered “acceptable” if the reference gene returns a ct between 29 and 40. Considering only the reference performance, a PCR reaction with 100% efficiency and y-intercept of 40ct would certainly allow you to use 1600 copies. If the intercept drops to 39.5 with 100% efficiency the 1600 copies would exceed a ct of 29 (below) thus would be considered an invalid sample. Did any of the ct’s from your studies with 1600 copy input show a reference below 29? If so, did you consider the samples as invalid? Same question for the cohort 1 and 2. Did any exceed the ct for the reference?
      You emphasize in your introduction the importance of detecting EGFR mutations occurring at less than 1% VAF. Again, not knowing the reference standard efficiency or y-intercept, I say the following with only assumption of how a PCR test normally functions, but if you claim that you can detect as small as 1 mutated copy and <1% is clinically important, then wouldn’t acceptable ct’s for the reference be too low when they are between ~35 – 40 to allow this?? Can you discuss what a reference ct of 40 means? Is the range of reference acceptance for the test too wide and would it allow for chances of underperformance for clinical use?
      Including not only the ct from the sample but the ct from the reference standard in the supplemental material for cohort 1 and 2 would be very useful to understand your data.
8) Discussion
   a) You properly conclude and describe the “possibility to detect as little as one copy” avoiding using the analytical term ‘limit of detection’ LoD should be limited to a proper assessment of the performance of an analytical test.
   b) You may wish to revisit of your summary that the dilution series display high linearity. Again they display a very high linear relationship between the measured and expected/anticipated value however you have not shown linearity across the entire 7 log scale. Please see CLSI guidance EP-06 for an example of the assessment of linearity in a clinical assay.
   c) Interesting discussion in the repeatability paragraph suggesting multiple replicates. Not all tests allow this due to sample input needs. Very good.
   d) You discuss the need for high specificity. I am unsure if you are referring to specificity as a term for precise selectivity based on the chemistry of your assay design or in the clinical sense as used to indicate a false positive rate. The SensiScreen biochemistry is impressive and it should lead to limited numbers of false reactions but this paper has not examined this, which to this reviewer is a deficiency that limits the observations of excellent sensitivity.
   e) Paragraph at line 502 “Evaluation using clinical liquid biopsy….”, you claim the test outperforms the other tests. For this statement to be of scientific merit please include a statistical assessment where significance of the statement is shared with the reader. What is the lower bound of th 95% CI for the agreement to Oncomine? Etc. Statements like “outperform” should be shown to have statistical relevancy. It may very well outperform, but without statistical assessment showing this then it is just the authors observation.
