# Supplementary material for: A new sensitive and fast assay for the detection of EGFR mutations in liquid biopsies
Source: PLoS One. 2021 Jun 24;16(6):e0253687. doi: 10.1371/journal.pone.0253687 (PMC8224962; doi:10.1371/journal.pone.0253687)
Supplement: Attachment — Submitted filename: Response to reviewers.docx [file pone.0253687.s010.docx]

**Additional Editor Comments:**

We would like to thank the editor for the time invested in reading our paper and for the precise indications that will permit to do changes in the submission and to meet PLOS ONE`s publication criteria.

Please refer to the attached file for the second reviewer's comments.

Journal Requirements:

When submitting your revision, we need you to address these additional requirements.

1. Please ensure that your manuscript meets PLOS ONE's style requirements, including those for file naming. The PLOS ONE style templates can be found at

<https://journals.plos.org/plosone/s/file?id=wjVg/PLOSOne_formatting_sample_main_body.pdf> and

<https://journals.plos.org/plosone/s/file?id=ba62/PLOSOne_formatting_sample_title_authors_affiliations.pdf>

 We verified the PLOS ONE`s style requirement and we changed the submission accordingly.

2. Thank you for stating the following in the Competing Interests section:

"Competing Interests:

UBC, RKP, MB, and CLM are employees of PentaBase ApS.

The SensiScreen Liquid EGFR assay is now part of a marketed product portfolio of PentaBase ApS."

We note that one or more of the authors are employed by a commercial company: PentaBase ApS.

We confirm that the aforementioned authors are employees of PentaBase ApS as stated in the competing interest statement.

2.1. Please provide an amended Funding Statement declaring this commercial affiliation, as well as a statement regarding the Role of Funders in your study. If the funding organization did not play a role in the study design, data collection and analysis, decision to publish, or preparation of the manuscript and only provided financial support in the form of authors' salaries and/or research materials, please review your statements relating to the author contributions, and ensure you have specifically and accurately indicated the role(s) that these authors had in your study. You can update author roles in the Author Contributions section of the online submission form.

Please also include the following statement within your amended Funding Statement.

“The funder provided support in the form of salaries for authors [insert relevant initials], but did not have any additional role in the study design, data collection and analysis, decision to publish, or preparation of the manuscript. The specific roles of these authors are articulated in the ‘author contributions’ section.”

If your commercial affiliation did play a role in your study, please state and explain this role within your updated Funding Statement.

The Financial disclosure statement has been modified (see attached file). The project was supported by EuroStars (project number E!8421). EuroStars did not have any additional role in the study design, data collection and analysis, decision to publish, or preparation of the manuscript.

PentaBase and ICP played the major role in study design, data collection, and preparation of the manuscript, as mentioned in the authors contribution. ICP is the scientific leader of the consortium. The role played by the different authors is reported in the “Authors contribution” section. The “Authors contribution” section has been implemented with the description of all the Authors involved in the present study.

2.2. Please also provide an updated Competing Interests Statement declaring this commercial affiliation along with any other relevant declarations relating to employment, consultancy, patents, products in development, or marketed products, etc.

The commercial affiliation of 4 co-authors has already been declared. The present research study was not influenced by any employment, consultancy, patents, products in development at PentaBase. Therefore, we do not have more information to add and we hope that our statement is acceptable

Within your Competing Interests Statement, please confirm that this commercial affiliation does not alter your adherence to all PLOS ONE policies on sharing data and materials by including the following statement: "This does not alter our adherence to  PLOS ONE policies on sharing data and materials.” (as detailed online in our guide for authors [http://journals.plos.org/plosone/s/competing-interests](about:blank)) . If this adherence statement is not accurate and there are restrictions on sharing of data and/or materials, please state these. Please note that we cannot proceed with consideration of your article until this information has been declared.

We confirm that the commercial affiliation of four people involved in the present research activity does not lead to any restriction on sharing data and/or material. As a consequence, we have added the sentence “This does not alter our adherence to PLOS ONE policies on sharing data and materials” to the competing interests statement (see attached file).

Please include both an updated Funding Statement and Competing Interests Statement in your cover letter. We will change the online submission form on your behalf.

An updated cover letter has been attached with the inclusion of the two statements (see attached document).

3. We note that you have included the phrase “data not shown” in your manuscript. Unfortunately, this does not meet our data sharing requirements. PLOS does not permit references to inaccessible data. We require that authors provide all relevant data within the paper, Supporting Information files, or in an acceptable, public repository. Please add a citation to support this phrase or upload the data that corresponds with these findings to a stable repository (such as Figshare or Dryad) and provide and URLs, DOIs, or accession numbers that may be used to access these data. Or, if the data are not a core part of the research being presented in your study, we ask that you remove the phrase that refers to these data.

As the data mentioned but not reported are not a core part of our study, therefore we have decided to remove the references to these data, according to PLOS ONE policies.

Reviewers' comments:

Reviewer's Responses to Questions

**Comments to the Author**

1. Is the manuscript technically sound, and do the data support the conclusions?

The manuscript must describe a technically sound piece of scientific research with data that supports the conclusions. Experiments must have been conducted rigorously, with appropriate controls, replication, and sample sizes. The conclusions must be drawn appropriately based on the data presented.

Reviewer #1: Yes

Reviewer #2: Partly

We have modified all the critical points raised by the reviewer 2 performing new experiments and using the correct terminology, thus hoping to receive a positive feed-back from him/her. New experiments required the involvement of a new institution, the Rigshospitalet/University of Copenhagen, that provided the samples from healthy donors and that helped us in the evaluation of the relative analyses. Therefore, we ask for the modification of the Board of the Authors, by adding Erik Sørensen, the person who did this work.

As a further consequence, the addition of new experiments and new pictures has lead us to modify the number of figures and tables in the manuscript as well as in the supplementary material.

2. Has the statistical analysis been performed appropriately and rigorously?

Reviewer #1: Yes

Reviewer #2: No

We have done the statistical analyses suggested by reviewer 2.

3. Have the authors made all data underlying the findings in their manuscript fully available?

The [PLOS Data policy](http://www.plosone.org/static/policies.action#sharing) requires authors to make all data underlying the findings described in their manuscript fully available without restriction, with rare exception (please refer to the Data Availability Statement in the manuscript PDF file). The data should be provided as part of the manuscript or its supporting information, or deposited to a public repository. For example, in addition to summary statistics, the data points behind means, medians and variance measures should be available. If there are restrictions on publicly sharing data—e.g. participant privacy or use of data from a third party—those must be specified.

Reviewer #1: Yes

Reviewer #2: Yes

4. Is the manuscript presented in an intelligible fashion and written in standard English?

PLOS ONE does not copyedit accepted manuscripts, so the language in submitted articles must be clear, correct, and unambiguous. Any typographical or grammatical errors should be corrected at revision, so please note any specific errors here.

Reviewer #1: Yes

Reviewer #2: Yes

5. Review Comments to the Author

Please use the space provided to explain your answers to the questions above. You may also include additional comments for the author, including concerns about dual publication, research ethics, or publication ethics. (Please upload your review as an attachment if it exceeds 20,000 characters)

Reviewer #1: 1. This study evaluated the performance of the newly developed and commercially available SensiScreen® EGFR Liquid kit for detection of the most diffused and clinically relevant EGFR mutations in cfDNA from NSCLC patients - (c.2369C>T (p.T790M), Exon 19 deletions, c.2573T>G & c.2573_2574TG>GT (p.L858R)) -, accounting cumulatively more than 90% of the mutations that can hyper activate EGFR. The results found that the SensiScreen® EGFR Liquid assay is able to identify down to 1 copy of mutant alleles and with its high sensitivity, linearity and accuracy it may be a competitive platform of choice.
2. Experiments, statistics, and other analyses are performed to a high technical standard and are described in sufficient detail.
3. Conclusions are presented in an appropriate fashion and are supported by the data.

We would like to thank the reviewer 1 for the positive evaluation of our study.

Reviewer #2: The authors should be commended on performing this study which defines the performance of a commercially available test which will be used to aid physicians in understanding the course of care for their patient. The strength of the paper is in the orthogonal testing done to support the clinical observations of the test in addition to the repeatability and challenge of the low end sensitivity of the test. The weakness in the paper is the lack of items in the analytical validation work, specifically in the lack of testing variant negative patient samples to establish the LoB and the clinical specificity of the test. This reviewer recommends that the authors follow the conventions of the analytical assessments of a clinical diagnostic with their use of terminology and how these are measured and displayed, such as LoD and linearity.
I would like to see the authors discuss a potential weakness of the assay in the wide allowance of amplifiable DNA used in the test and how this wide window may result in inconsistent clinical assessments of the EGFR mutation.
Please add some statistical test to groups you display in graphs and also add ranges to your data and 95% CI as necessary.
Overall it is recommended that this paper be published in PLoS One as it is important that assessments of tools being used by clinicians be properly described and compared.
Please see attached review.

At first, we would like to thank the reviewer 2 for the precise review, the very helpful comments and suggestions, and for the substantial positive evaluation of our study. We also thank him/her for his/her recommendation to publish our study in PLoS One. We have recruited a batch of negative samples (collected from healthy donors) and made experiments in line with reviewer’s suggestions. Furthermore, we have improved the statistical analyses and adapted the paper consequently. Please see below our detailed point-by-point reply.

6. PLOS authors have the option to publish the peer review history of their article ([what does this mean?](https://journals.plos.org/plosone/s/editorial-and-peer-review-process#loc-peer-review-history)). If published, this will include your full peer review and any attached files.


If you choose “no”, your identity will remain anonymous but your review may still be made public.


**Do you want your identity to be public for this peer review?** For information about this choice, including consent withdrawal, please see our [Privacy Policy](https://www.plos.org/privacy-policy).

Reviewer #1: No

Reviewer #2: No

[NOTE: If reviewer comments were submitted as an attachment file, they will be attached to this email and accessible via the submission site. Please log into your account, locate the manuscript record, and check for the action link "View Attachments". If this link does not appear, there are no attachment files.]

**Comments in the review of Jensen et al, A new sensitive and fast assay for the detection of EGFR mutations in liquid biopsies**

The authors have presented a paper which is important in further defining performance of the tools available for aiding physicians in applying targeted therapy to their patients. This paper explores analytical measures allowing the end user a way to understand how the test performs and to be able to assess how the test compares to other options an end user has available. In defining the performance of the test, the authors explore analytical measures such as limit of detection, linearity, repeatability and accuracy as approached through orthogonal comparison to two other ctDNA platforms. This paper follows the content of Riva et al, SensiScreen® KRAS exon 2-sensitive simplex and multiplex real-time PCR-based assays for detection of KRAS exon 2 mutations, PLoS One 2017 and the reviewer recognizes that the Riva paper’s approach and presentation has been accepted prior. However, there are some items which this reviewer feels need further consideration by the authors. The comments and suggestions are offered in order to harmonize the terminology in describing the analytical performance of diagnostic tests thereby allowing physicians/readers who are assessing testing methods as published in peer reviewed journals to see measurements based on clear and consistently applied definitions of analytical performance.

1) General comment: With your data, please include appropriate errors or ranges within the data you present. For instance, with your first plot showing the age of the patients in cohorts, what is the mean, range and did you apply a statistical measure to show they are the same? For the dilution series, what is the CV or SD experienced at each dilution point. Etc.

In agreement with the reviewer’s suggestion, we have now included a table in Fig1 containing the descriptive information of the data, such as size of each cohort, median, range and standard deviation. Furthermore, as suggested by the reviewer, we have performed a comparative test between cohort I and II. No differences in age distribution were found (p= 0.23). The information are now included on the manuscript, in material and methods, Figure 1 has been revised (page 7 line 138-139, Figure 1). The average and SD of the dilution series have been reported in Table S4, S5, S6 and in Figure 2, 4b.

2) In the use of contrived material to define the analytical performances of the test the authors did not indicate that fragmented DNA was utilized in these experiments. If fragmentation was used, please indicate the process (sonication, restriction enzymes etc) and mention or perhaps show in the appendix that your fragmentation procedure produced a range of fragments somewhat similar to what is found from cfDNA. This relates to both the cell line material and the Promega mixed human genomic DNA which is sold as having >90% of the DNA as longer than 50kb in size.

If the authors did not utilize fragmented DNA then they should defend why this diagnostic approach (SensiScreen) would not be impacted by random fragmentation which could break DNA between the For and Rev primers thereby allowing the proper non-binding of the BaseBlocker in a fragment with the mutation and allowing binding of the For primer but extension stopping prior to where proper binding of the Rev primer would occur. This is important in the claims being made in the paper regarding the analytical sensitivity of the diagnostic system. Assuming no fragmentation of your cell line DNA/human DNA, are there other impacts on the PCR reaction which may be influenced by larger vs fragmented DNA besides the random fragmentation within the amplicon region? Ideally a contrived analyte should mimic as best as possible the biological analyte and if this is not important please defend why the authors chose to this route.

ctDNA from plasma is highly fragmented, with an expected maximum size of about 140-150 bp. Very often, fragments size are even shorter. To have, as the reviewer suggests, a similar size of the DNA used in the analytical sensitivity experiments, we should nearly completely destroy the DNA. We decided not to do any fragmentation because our aim was to estimate the precise sensitivity of our assay under reproducible conditions, whereas the analysis of true plasma samples from patients is subjected to many variables and may provide different results depending on the batch that is under evaluation. Therefore, for the experiments of sensitivity, we evaluated unfragmented genomic DNA and we verified that our assays are able to identify down to one copy of the analyte. Translated in true plasma samples, it means that our assay should be able to identify down to one intact mutant allele.

In addition, the fragmentation of DNA could be evaluated in our assay by the HEX signal (internal control) values: we did not see significant differences between the samples used for the definition of analytical performances and the cfDNA obtained from plasma samples of the patients.

The Authors utilize the term LoD (limit of detection) in a manner which is not the acceptable application of this term in the description of the analytical limits for a clinical assay (for example CLSI-EP17A or IUPAC Recommendations (L Currie, Nomenclature in evaluation of analytical methods including detection and quantification capabilities (IUPAC Recommendations 1995) ACA 391, 105-126).

a. For assessing the assay’s background/noise, the authors used only one sample (WT genomic DNA). With the lack of using age matched normal donors (samples assumed to not have the mutation present) with DNA isolated from plasma in describing the background (limit of blank) for each variant in the assay, the readers to cannot be assured that the claim of being able to detect 1 copy is in actuality the clear detection of the signal arising from one copy of the target DNA above what would be experienced with samples without the mutation tested under the same conditions (input level etc).

We performed new experiments on DNA from 58 age-matched healthy donors. Please see the updated paper for the specific results that confirmed the data included in the previous version of our manuscript: the results are reported on page 16 lines 350-353 and shown in Figure 5 (A, B and C).

Authors should supply evidence supporting the limit of blank and also provide the limit of detection as defined by guidelines. Often this is defined in relationship to LoB and where 95% of the measurements is a repeated sample fall above the LoB. It can be acceptable to defend the success rate at a lower value, ie LoD90, but it should be clear to the reader what definition is used for LoD.

We modified the terminology properly. The performed LoB studies revealed that when sticking to the guidelines recommendations concerning template load, 0% of the 20 repetitions for all mutation assays resulted in false positive results. Only 4 out of the samples analyzed gave rise to Ct values with the lowest registered value being 41.77, close to two cycles above the limits set for the assay.

Should the authors move away from the use of the term LoD and describe this as something like the “lowest value detectable” by the assay system, the lack of evidence from the testing of samples/donors without the given mutation raises questions as to whether or not the detection of 1 copy is limited to only samples/patients who carry the given variant and should be addressed.

In the updated version of the paper we used the terminology properly (e.g.: see page 24).

3) The above discussion on LoB leads to another deficiency in this paper and that is in establishment of the specificity of the test. It was shown in the post-hoc analysis of the data from the gefitinib pivotal trial by Mok et al (NEJM 2009) that the very worst outcome for a patient being considered for EGFR TKI usage or for SOC-doublet chemo, that a false positive EGFR mutation call would lead the patient to not receive doublet therapy and be put on TKI therapy thereby receiving a therapy with a detrimental survival outcome. The authors should consider running a set of real plasma samples (age matched, if possible at the same DNA input levels as your contrived cell line dilutions) to both get a sense of test specificity and to establish the LoB, thereby defending that your ability to detect down to 1 copy is well above any noise in the system. Running a single wt Promega DNA mixture is not sufficient for this.

We agree with the comments of reviewer 2 and to clarify this point we ran plasma from 58 age-matched (from 36 to 68 years old) healthy donors. We have not detected any mutant allele in these plasma samples, thus indicating that our assays should not provide false-positive cases. The results are reported on page 16 lines 350-353 and shown in Figure 5 (A, B and C).

In cohort I which describes the use of Streck BCT tubes, the authors refer to serum in addition to plasma. It is unacceptable for blood being processed to serum to be held for 48 hours prior to separation as leukocyte degradation and subsequent release of leukocyte genomic DNA has been shown to occur. Please clarify if serum is the correct term here and if it was, further description of the use of serum tubes and a discussion on the impact of contaminating genomic DNA should be offered by the authors.

We completely agree with reviewer 2, we made mistakes in the description of the methodology. Concerning this point, we added a few statements concerning serum collection and separation (see page 10 lines 212-219).

5) Results section
   a) Sensitivity – please provide the CV or SD at each dilution point.

The average and SD of the dilution series have been reported in Table S4, S5, S6 and in Figure 2, 4b.

b) Linearity – R2 describes the relationship between two measured variable and the strength of the relationship. 0.97 means that you show a very good linear relationship between ct and the estimated copy number but you do not show linearity as have referred to this as. It would be more appropriate to apply other tests of linearity to your dilution experiment if you wish to claim or show that your test has linearity. I would recommend that you read CLSI guidance EP-06. Applying an assessment of a 1^st^ order polynomial fit to this data to a second or third order polynomial fit will show where the data is linear and where the linearity drifts.

Linearity data has been introduced in S3 Table, cited at page 14.

c) Repeatability – Section well written and described. Please describe the data further as you see fit. Example mean, SD, 10^th^ and 90^th^ percentile etc.

We updated the manuscript describing data with statistical evaluation of fractile limits which have been chosen according to theoretical number of zero samples and of samples with more than 1 copy assuming a concentration of 1 copy/5 microL and Gaussian distribution (please see page 16 lines 343-346 and S6 Table).

6) SensiScreen in clinical samples
   a) You give median DNA/ml extracted, please include 10^th^ and 90^th^ percentile or range in the text.

We would like to thank reviewer 2 for highlighting this important point which also did that we explored that the serum concentrations by a mistake was included in the median value for cohort I at first. We apologize for this and have now changed the median concentration for Cohort I including only the plasma samples, and added the DNA/ml ranges for both Cohorts for plasma samples (please see page 18 lines 378-382 and Figure 6).

b) To follow on from a comment above regarding serum in cohort 1, I see you have 3 serum samples. Here or later in the discussion if appropriate, you may wish to comment that the DNA/ml isolated from the serum was within the range of the Streck plasma suggesting that a gross contamination from DNA released from leukocytes was not observed.

We would like to thank reviewer 2 for highlighting this relevant point, we have added this consideration in the results section and modified the text accordingly (see page 18 lines 383-385).

c) For data in figure 4 please perform a statistical test to compare the ng cfDNA from Streck vs citrate. It appears that citrate may give a statistically higher amount of DNA. Please test if the two sets of isolated DNA are different from one another. One might assume that there is a release of HMW DNA in the citrate cohort and you should discuss if this HMW DNA could have an impact on the assessment of the mutations by any methodology. Worth considering this in your discussion should they show a statistical difference.

As well-indicated by the reviewer, we have performed the comparison between DNA concentration from both tubes. In line with the reviewer’s assumption, a difference was observed between those two extraction methods (p= 0.021), with citrate yielding higher amounts of DNA, as depicted on the Figure S1D. We have now included that information as Supplementary Figure S1D, and included the result in the discussion (pages 23-24 lines 461-466).

For Table 1A and 1B it is rather unusual to see T790M without the 19del or other driver such as L858R. This indicates that the patient has an inherited T790M (unusual but does occur) or that the driver is not seen due to the tests chosen not having the designs for the driver mutation. There are 5 total examples of this. I see that you discuss this example “c”. You are saying that EntroGen detected this sample as ex19del positive but T790M negative and SensiScreen detected T790M but not the ex19Del. This is biologically very unusually as T790M is a branch variant from the ex19del truncal variant. Ex19del is observed in quantitative tests as having a higher VAF than the acquired mutation.

We are conscious that it is unusual to see the presence of T790M without other driver mutations such as 19del or L858R, however these results are confirmed by all the methodologies that we applied in our study (and we also observed it in other diagnostic patients, not included in the present work – Milo Frattini’s personal data).

Using table S3b, for the other examples where T790M is detected without the driver detected, can you describe why this might have occurred?

In some cases the T790M can occur as primary mutation, these could be the case of the samples that in Table S3b do not show other driver mutations. From what we have experienced from our clinical setup, we are indeed of this opinion that we for, though few, some samples observed T790M as the dominant primary mutation as evaluated from the relative high expression observed for T790M. This could be due to the effect of the treatment administrated to the patients which may have killed and thereby lowered the number of tumor cells containing the driver mutation to a minimum where the driver mutation is not detectable in blood.

Is or could the ex19del be an unusual deletion which SensiScreen did not have a design for?

Ex19 del PentaBase assay is designed to identify all the deletions that involve codons 746-748: if the deletion is completely out of this region, it cannot be identified (for example, the deletion 751-757 is wt for PentaBase assay). However, we have to mention that the mutations that do not involve the 746-748 region are a small subgroup.

How do you defend not seeing the drivers from patient 25,26 and 27? What is the ct of the T790M for these samples? Are they just at the 1 copy area? Including the ct values in the supplemental tables would help a reader to further understand your observations.

Concerning patients 25, 26 and 27 the disappearance of driver mutations from analysis of tumor tissue months to years prior to ctDNA analysis, using both methodologies (Entrogen and SensiScreen), could be due to the effect of the treatment administrated to the patient. Indeed the treatment could have been more effective on clones containing the driver mutations permitting a predominance of clones characterized by the T790M. Alternatively, the driver mutation can be lost due to gene/chromosomal rearrangements of the mutant allele, while the T790M (which should be *in trans*) is maintained and determines cellular fate.

We decided not to add ct values of the analyzed cohort in a supplemental table because this datum has never been reported in any study on this topic in the literature.

In Table S3A and S3B it would be ideal if you also included the measured values (ct, VAF, copy number; whatever is the usual output) for all liquid biopsy tests.

VAF values have not been included for the same reason of the last point.

d) In Table S3A, you should identify the construct of the 19del detected in Oncomine as 19del comes in many presentations. In Table S3B, it is unclear what tissue test was used to confirm the variants, however if it is available, please include the construct of the 19del. It is important to know if there is a design available for the specific ex19del in the liquid biopsy tests.

PentaBase assay for the analysis of exon 19 deletions enables the identification of all the deletions involving codons 746-748, but is a multiplex assay that cannot differentiate among the different deletions. Due to the structure of the Danish health society, the tissue tests were performed in different laboratories using different – for us unknown - validated tests so we do not have the possibility to include this for most of the samples.

e) Patient 13 cohort II was WT in tissue and now has only T790M in both liquid assays. Was this patient treated with 1^st^ or 2^nd^ gen TKI? Why were they treated with TKI if no evidence of EGFR driver mutation? How did a T790M arise?

The study performed focus on method comparison. It is not possible to retrieve treatment information from the individual patient. We are not allowed to look up in patient records for treatment, but we agree on that it not clinically relevant to treat (standard guidelines) WT patients with TKI. A reason for TKI treatment could be that the patient was included in a clinical trial or in an experimental setting with TKI treatment based on mutations in other genes, thereby the T790M may have been introduced. This has now been included in Table S7B.

f) For table 1A and 1B, in accurate NGS testing, the EGFR driver is nearly always found at greater VAF than the acquired T90M mutation. It is important that when you compare the two tests that you do discuss the impact of different DNA amounts if the DNA amounts for the testing was limited due to the need to split the samples up for this study. Your discussion regarding the ex19 variant detected only at higher levels of DNA input for EntroGen vs SensiScreen is a good example of detection sensitivity vs sensitivity with increasing input.

The finding that the VAF of EGFR driver mutation is greater with the respect to the one of T790M can be explained by the fact that not all the cells with the EGFR driver mutation carries the T790M change.

Concerning the hypothetical different DNA input, our study is a retrospective study in which SensiScreen assays have been tested for validation on true plasma samples previously characterized, for diagnostic level, by other methodologies. Therefore, NGS or EntroGen assays were applied under the best technical conditions. But the same is true also for SensiScreen assays, because cases from our archives for which a limited amount of plasma samples was available, were discarded.

g) Table IB, footnote (b), and paragraph starting on line 394 “For cohort II, as expected from the results….” In order to justify your conclusion more clarification is necessary. You say that SensiScreen only had limited material hence the T790M dropped out but you wish to consider these as “in agreement” I need a bit more information before I would be in agreement. It appears to be a discordance which results in dire clinical guidance. For prescribing osimertinib after 2^nd^ gen EGFR TKI, one must have a T790M mutation and these results with SensiScreen deny the patient the chance to start on osimertinib. My question is regarding the limited material: did the amount used in this sample pass the reference gene QC requirement for the test? I believe this is a ct of the reference to be between 29 and 40 as I read in the “SensiScreen, instructions for use”. Or did it show lower than a 40 ct? If yes, it was lower than the low limit in the package insert I can see your argument and I would describe it this way. However, if the reference passed QC / deemed adequate, and thus the sample was acceptable, there is a need to discuss this beyond “we consider them in agreement”. Should the manufacturer allow for a very low level of amplifiable DNA in the test or DNA input for the test, this should be a critique in your discussion part of your paper that a test can be valid but allowable levels my limit the detection to up to 10% VAF or such. By not having a critical lower limit of input, a doctor relying on the results of this test for aiding patient care would provide inconsistent patient care.

We agree with reviewer 2 that seen from a clinical point of view this should be seen as disagreement samples, but as our aim of this study is to perform a method comparison study - of course with the overall aim to use the best possible platform from our study for clinical decisions regarding treatment of the patients – they shall be seen as agreement samples. Unfortunately, the reason why there is very few studies comparing different platforms for liquid biopsy testing in clinical setups is that the amount of liquid biopsy material (here DNA) is very limited, and often the majority of DNA, if not all, will be used for the initial clinical analysis. As also the case in our study, this means that researchers may have fewer microliters left for second platform analysis. As such, after comparing our results obtained for both platforms, we could see - from our initial clinical analysis of sample 21 - that the sample material input for the Pentabase platform evaluation was not enough for detection of EGFR T790M, but still enough for detection of the Exon 19 del (25ng ~ 1-3 copies of T790M vs. 5ng ~ below 1 copy of T790M). The same was observed with regard to sample 22 (23 ng ~ 1-2 copies of T790M vs. 10ng below 1 copy of T790M) after comparison. Therefore, after completed testing of both platforms we could conclude that the angle of entry for the evaluation of the Pentabase platform for those samples was that the PentaBase platform should not find both mutations as present at input amounts used for the Entrogen platform. They are therefore agreement samples under the given circumstances.

h) To expand further on the reference measurement above, another important consideration in your data is following the limits set by the manufacturer for the test. Part of the sample QC is in the use of a reference target which “is used to assess the amount of amplifiable DNA in the sample”. A sample is considered “acceptable” if the reference gene returns a ct between 29 and 40. Considering only the reference performance, a PCR reaction with 100% efficiency and y-intercept of 40ct would certainly allow you to use 1600 copies.

If the intercept drops to 39.5 with 100% efficiency the 1600 copies would exceed a ct of 29 (below) thus would be considered an invalid sample. Did any of the ct’s from your studies with 1600 copy input show a reference below 29? If so, did you consider the samples as invalid? Same question for the cohort 1 and 2. Did any exceed the ct for the reference?
You emphasize in your introduction the importance of detecting EGFR mutations occurring at less than 1% VAF. Again, not knowing the reference standard efficiency or y-intercept, I say the following with only assumption of how a PCR test normally functions, but if you claim that you can detect as small as 1 mutated copy and <1% is clinically important, then wouldn’t acceptable ct’s for the reference be too low when they are between ~35 – 40 to allow this?? Can you discuss what a reference ct of 40 means? Is the range of reference acceptance for the test too wide and would it allow for chances of underperformance for clinicaluse?
Including not only the ct from the sample but the ct from the reference standard in the supplemental material for cohort 1 and 2 would be very useful to understand your data.

In general for PCR, and especially when using PCR for liquid biopsy testing, you will not have a 100% efficiency of your PCR at the time point for detection of the low copy variants in your clinical sample material. There are more both intra- and inter sample factors that may influence your PCR. First, when samples are collected they are often collected by different people with different experience level. It is a well-known phenomenon that 3 different people may execute a SOP as blood collection and preservation before DNA extraction in different ways leading to variations introduced even before DNA extraction (Contamination from blood cells making the DNA background extreme in order to detect the few copies that may be available with a Real-Time PCR – here you may for such samples succeed with a Droplet digital PCR instead). Next, during DNA extraction you will also likely introduce some intra- and inter sample factors that may influence your PCR (Inhibitors etc.). Next, total cfDNA concentrations are measured and is often very low meaning that there also here are some wide uncertainty regarding the exact amount that you actually spike into your PCR. But of course, here you have the QC acceptance criteria (within Ct 29-40) for your reference as a measure for that you are within the acceptance criteria stated by the manufacturer. But still, it is important to take all possible factors that may have an impact on your PCR into consideration when evaluating your result – and not only the strict PCR theory. And yes, the intra- and inter sample factors (i.e. inhibitors) may do that you can actually have two samples with equal DNA concentrations and same DNA input for your PCR that shows equal Cts at detection for the references (because the difference of inhibitors will not impact the PCRs for the references thereby showing no difference here), but still have an extreme impact on the prevention of detection of the few target copies present in your sample with the highest amount of inhibitors. The efficiency of your PCR will here vary between the two samples meaning that you may detect the few copies of your target in the sample with less inhibitors as compared to the other sample with a higher concentration of inhibitors. Therefore, in the clinical setup you need to take all of the different steps into consideration and see the QC reference criteria as stated by the manufacturer in the light of this. As such they should of course be used, but as an overall QC guidance according to all other factors. The above mentioned reasons and explanation is also the reason why the Ct values are obtained at time points where we will not expect that the PCR efficiency to be 100% and we acknowledge that it might be of interest to some readers but overall and due to the variable factors mentioned above we are not of the opinion that Ct values should be added to the manuscript as they may be interpreted as more exact values (100%) than they should be by more potential readers. It is always important to be aware of how exact/strict your results should be interpreted taking everything including the method into consideration. And liquid biopsy is an especial good example of this. In our clinical setup we have for more of the samples performed triplicate analysis and observed that the detection of only two out of the three triplicates meaning that the PCR efficiency indeed is varying critical at low target amounts.

7) Discussion
   a) You properly conclude and describe the “possibility to detect as little as one copy” avoiding using the analytical term ‘limit of detection’ LoD should be limited to a proper assessment of the performance of an analytical test.

We would like to thank reviewer 2 for this precise definition and therefore we modified the text accordingly

b) You may wish to revisit of your summary that the dilution series display high linearity. Again they display a very high linear relationship between the measured and expected/anticipated value however you have not shown linearity across the entire 7 log scale. Please see CLSI guidance EP-06 for an example of the assessment of linearity in a clinical assay.

We have evaluated the dilution points in the entire 7 log scale and they present linearity, also following the CLSI guidance EP-06 guidelines. We have not reported the data in the paper because the description could be redundant.

Interesting discussion in the repeatability paragraph suggesting multiple replicates. Not all tests allow this due to sample input needs. Very good.

We would like to thank reviewer 2 for this comment.

c) You discuss the need for high specificity. I am unsure if you are referring to specificity as a term for precise selectivity based on the chemistry of your assay design or in the clinical sense as used to indicate a false positive rate. The SensiScreen biochemistry is impressive and it should lead to limited numbers of false reactions but this paper has not examined this, which to this reviewer is a deficiency that limits the observations of excellent sensitivity.

We agree with reviewer 2 that it is very important to be precise in terminology, also when talking about specificity. Here, by writing specificity, we mean specificity according to the methods ability to exclude false positive detection by not amplifying DNA from the big pool of background DNA not related to the, in comparison, relative few DNA targets of clinical interest. In line with the reviewer 2 comment, we are of that opinion, if our aim of the paper also had been to evaluate the specificity according to the chemistry behind the SensiScreen a further in depth explanation of the term specificity had been necessary.

d) Paragraph at line 502 “Evaluation using clinical liquid biopsy….”, you claim the test outperforms the other tests. For this statement to be of scientific merit please include a statistical assessment where significance of the statement is shared with the reader. What is the lower bound of the 95% CI for the agreement to Oncomine? Etc. Statements like “outperform” should be shown to have statistical relevancy. It may very well outperform, but without statistical assessment showing this then it is just the authors observation.

We added and updated statistical analyses in the paper and Tables.
